# Comparison of Sleep Quality Across Different Sagittal Skeletal Patterns in Orthodontic Patients

**DOI:** 10.3390/healthcare14203489

**Published:** 2026-10-17

**Authors:** Chanistha Sirichan, Narissaporn Chaiprakit, Siripatra Patchanee

**Affiliations:** 1 Division of Orthodontics, Faculty of Dentistry, Thammasat University, Pathum Thani 12120, Thailand; chanistha.sir@gmail.com; 2 Division of Oral and Maxillofacial Surgery, Faculty of Dentistry, Thammasat University, Pathum Thani 12120, Thailand; nchaiprakit@gmail.com

**Keywords:** sleep quality, Pittsburgh Sleep Quality Index, skeletal patterns, maxillomandibular relationship, orthodontics

## Abstract

**Highlights:**

**What are the main findings?**

• 55.9% of the orthodontic patients in this study reported poor subjective sleep quality.
• No significant differences in subjective sleep quality were observed among Skeletal classification I, II, and III groups.

**What are the implications of the main findings?**

• Anteroposterior maxillomandibular relationship classification alone may be insufficient to identify orthodontic patients with poor sleep quality.

**Abstract:**

**Background/Objectives:** Sagittal skeletal patterns are associated with upper airway dimensions, which may influence breathing during sleep and potentially relate to sleep quality. We hypothesized that global Thai-Pittsburgh Sleep Quality Index (Thai-PSQI) scores would differ among skeletal Class I (Sk-I), Class II (Sk-II), and Class III (Sk-III) groups. **Methods:** This cross-sectional study was conducted at the Department of Orthodontics, Faculty of Dentistry, Thammasat University, from June 2023 to March 2024. Ninety-three patients aged 18–50 years presenting to initiate orthodontic treatment were classified by ANB angle as Sk-I (0.5–5.1°), Sk-II (>5.1°), or Sk-III (<0.5°), with 31 participants per group. Sleep quality was assessed using the Thai-PSQI; scores > 5 indicated poor sleep quality. Sex, age, and BMI were analyzed as demographic variables. **Results:** Mean global PSQI scores were 6.42 ± 2.71, 6.68 ± 3.39, and 6.26 ± 2.46 for Sk-I, Sk-II, and Sk-III, respectively. Global PSQI scores did not differ significantly among skeletal groups (H = 0.024, *p* = 0.988, ε^2^ < 0.001). Overall, 55.9% of participants were poor sleepers, and the distribution of good and poor sleepers did not differ among groups (χ^2^ = 1.134, *p* = 0.567, Cramer’s V = 0.110). No significant associations were observed between global PSQI and sex, age, or BMI (*p* = 0.279, 0.848, and 0.948, respectively). **Conclusions:** No statistically significant differences in subjective sleep quality were identified among the three sagittal skeletal groups in this sample.

## 1. Introduction

Sleep is an essential component of human health and daily functioning. Healthy sleep is associated with physical, mental, and cognitive well-being [1,2]. In contrast, poor or disrupted sleep is associated with adverse cognitive, psychological, and physical health outcomes [2]. The Pittsburgh Sleep Quality Index (PSQI) is a widely used, standardized, self-administered questionnaire that assesses sleep quality over a one-month period [3]. It is employed across clinical and non-clinical populations, including individuals with and without medical or psychological conditions [4,5]. The PSQI is used in clinical practice as a screening tool for sleep dysfunction [4,5] or to assess sleep quality in healthcare professionals [6], among other applications.

Skeletal relationships are commonly evaluated using cephalometric analysis to assess maxillomandibular discrepancies by orthodontists before treatment [7]. The ANB angle (A point-Nasion-B point) [8] and the Wits appraisal (AO-BO) [9] which are widely used to classify sagittal skeletal relationships into three categories: skeletal Class I (Sk-I), Class II (Sk-II), and Class III (Sk-III) [8,9,10]. The anteroposterior positions of maxilla and mandible, which contribute to the facial skeletal pattern, may influence upper airway dimensions [11,12,13]. Since the pharyngeal musculature is closely tied to the bone structure, these facial skeletal patterns may be associated with upper airway characteristics or abnormalities [14].

Orthodontists have also shown interest in the airway, as it plays a vital role in craniofacial development and is relevant to diagnosing and managing conditions such as mouth breathing and sleep disorders [15]. In addition, current evidence indicates that breathing disorders such as obstructive sleep apnea (OSA) are associated with diminished upper airway dimensions. The oropharynx is the most common site for airway obstruction [16,17]. Previous studies have reported that the posterior airway space and oropharyngeal volume decrease from Sk-III to Sk-I and then to Sk-II [16,18]. The nasal passage volume showed a different pattern, decreasing from Sk-I to Sk-III and then to Sk-II [16]. Moreover, a previous study found that micrognathia and skeletal class II relationships were the most common characteristics in retropalatal and retroglossal obstruction patients, but the retropalatal obstruction group had a lower incidence [19]. Previous studies have reported associations between sagittal skeletal patterns and upper airway dimensions [16,18], with a greater ANB angle associated with a significantly smaller oropharyngeal area [20]. Vertical skeletal pattern may also be associated with upper airway dimensions, with reduced oropharyngeal volume reported in hyperdivergent individuals. However, recent meta-analytic evidence suggests that sagittal skeletal differences involve several upper airway compartments, whereas statistically significant differences related to vertical pattern were primarily identified in the oropharyngeal region [21].

Narrowing of the pharyngeal structure may increase the tendency of pharyngeal collapse during sleep when the pharyngeal dilator muscles fail to generate sufficient force to resist the negative inspiratory pressure in the pharynx [22,23,24], potentially disrupting breathing during sleep. A recent study of adults seeking orthodontic treatment found no significant differences in global PSQI scores among Sk-I, Sk-II, and Sk-III groups, although sleep quality differed according to other craniofacial characteristics, including vertical skeletal pattern and hyoid position [25]. Nevertheless, evidence remains limited, especially among populations with different craniofacial features.

These findings together suggest that sagittal skeletal morphology may be relevant to sleep through its relationship with upper airway anatomy, although anatomical differences may not necessarily be reflected in subjective sleep quality. Sagittal skeletal pattern was predefined as the primary skeletal dimension of interest in this study. Accordingly, we aimed to compare subjective sleep quality among Thai orthodontic patients with Sk-I, Sk-II, and Sk-III using the Thai-PSQI. The primary outcome was the global Thai-PSQI score, and we hypothesized that it would differ among the three skeletal groups.

## 2. Materials and Methods

### 2.1. Study Design and Participants

**Figure 1:**
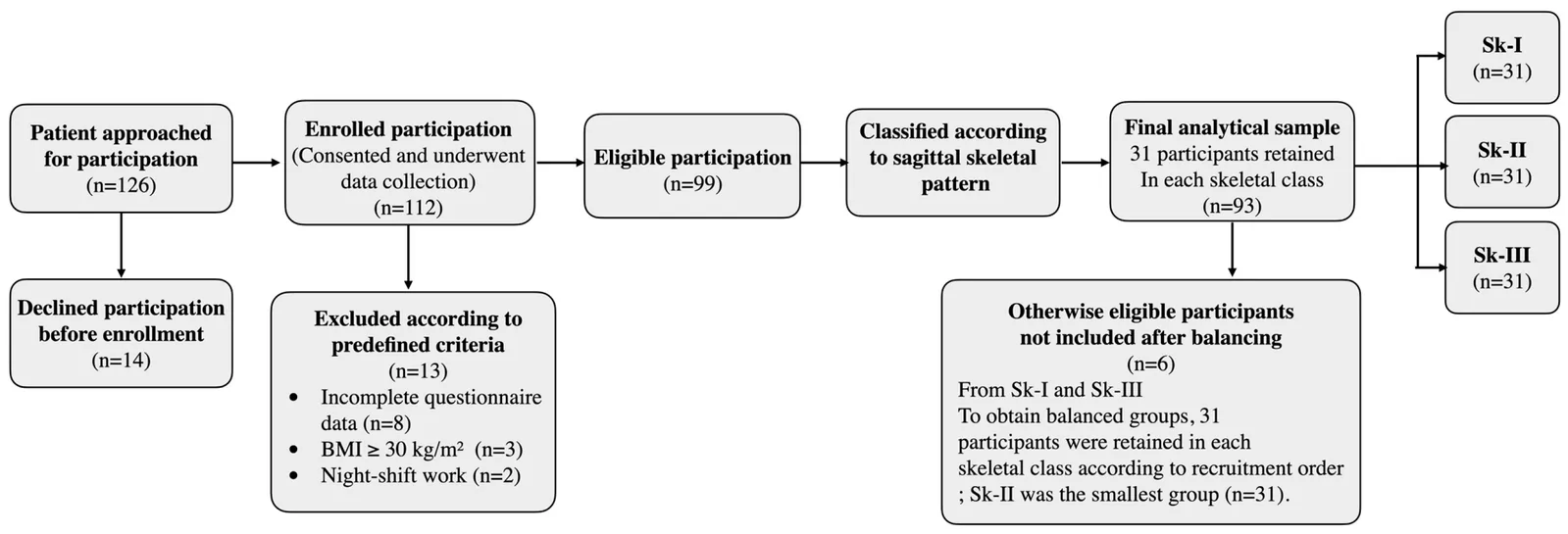
Flow diagram of participant recruitment and selection.

This study was conducted in accordance with the Declaration of Helsinki and approved by the Human Research Ethics Committee of Thammasat University (Science), Thailand (protocol code 66DE020; approved on 9 June 2023). This cross-sectional study was conducted at the Department of Orthodontics, Faculty of Dentistry, Thammasat University, between June 2023 and March 2024. Patients presenting to the department to initiate orthodontic treatment were approached consecutively for participation. Those who agreed to participate provided written informed consent, completed the PSQI questionnaire, and underwent cephalometric radiography for skeletal classification, all before the initiation of orthodontic treatment. An a priori sample-size calculation using G*Power version 3.1.9.6, based on data from a previous study [26], indicated that a minimum of 51 participants was required (effect size *f* = 0.44864, α = 0.05, power = 80%). A total of 126 patients were approached for participation, of whom 14 declined before enrollment and data collection. Among the 112 enrolled participants, 13 were excluded, leaving 99 participants who met the eligibility criteria and had complete data. Following skeletal classification, Sk-II was the smallest group (n = 31); therefore, 31 participants from each skeletal class were included in the final analysis to ensure balanced group sizes. For skeletal Sk-I and Sk-III, participants were included according to their order of recruitment. Thus, 93 participants were included in the final analysis (Figure 1). The final study sample comprised 93 Thai patients aged 18–50 years (32 males and 61 females). Participants had reached cervical vertebral maturation (CVM) stage CS6 and were proficient in reading, writing, speaking, and listening in Thai. The exclusion criteria were obesity [body mass index (BMI) ≥ 30 kg/m^2^], night shift work, a history of orthognathic surgery or orthopedic treatment in the craniofacial region, chronic respiratory disease, previous tonsillectomy and/or adenoidectomy, craniofacial trauma or pathology in the study area, a history of head and neck radiation therapy, self-reported mental or psychiatric disorders, and a self-reported history of physician-diagnosed obstructive sleep apnea.

### 2.2. Measures

Lateral cephalometric radiographs were taken with subjects in an upright position with the x-ray tube perpendicular to the sagittal plane and all subjects in the Frankfort horizontal plane parallel to the floor. During image acquisition, the head was stabilized using ear rods positioned in the external auditory meatus. Participants were instructed to bite in maximum intercuspation and inhale through the nose during image capture [27].

Cephalometric analyses were conducted using “Dolphin Imaging Version 11.95.08.50 Premium” (Dolphin Imaging and Management Solution, Los Angeles, CA, USA) to classify participants into Sk-I, Sk-II, and Sk-III groups using the Thai norm of ANB Angle (2.8° ± 2.3°) [25]. To classify the sagittal skeletal pattern, SNA, SNB, and ANB angle were measured: Sk-I, 0.5° ≤ ANB ≤ 5.1°; Sk-II, ANB > 5.1°; and Sk-III, ANB < 0.5°. All participants were assessed for SN length (males, 70.2 ± 4.6 mm; females, 63.4 ± 3.1 mm) and SN-FH angle (6.9° ± 3.1°) using Thai reference values [28]. The reference ranges used in this study were defined as the reference mean ± 1 SD, corresponding to 65.6–74.8 mm for SN length in males, 60.3–66.5 mm in females, and 3.8–10.0° for the SN-FH angle. If either measurement fell outside its reference range, ANB-based skeletal classification was confirmed using Thai Wits appraisal reference values (males, −0.6 ± 3.1 mm; females, −1.8 ± 2.6 mm) [28]. Using the same ±1 SD criterion, Wits values from −3.7 to 2.5 mm in males and from −4.4 to 0.8 mm in females indicated Sk-I. Values above the upper limit indicated Sk-II, whereas those below the lower limit indicated Sk-III. Wits appraisal was used to confirm skeletal classification in 18 of the 93 participants. In all 18 participants, the Wits-based classification agreed with the ANB-based classification. The Frankfort–mandibular plane angle (FMA) was also measured as a vertical skeletal parameter.

Nineteen cephalometric radiographs (20% of the total sample) were randomly selected and re-measured by the same examiner two weeks after the initial assessment to assess intra-examiner reliability. The examiner was blinded to the participants’ PSQI results during both assessments. Reliability was evaluated using a two-way mixed-effects, absolute-agreement, single-measure intraclass correlation coefficient (ICC) with 95% confidence intervals. The ICC values ranged from 0.982 to 0.998 (95% CIs, 0.955–0.999), indicating excellent intra-examiner reliability based on the predefined criterion of ICC > 0.90 [29]. Therefore, the measurements from the initial assessment were used for subsequent analyses.

The PSQI is a validated instrument designed to assess subjective sleep quality of patients and distinguish between good and poor sleepers [3]. The PSQI has been translated into multiple languages, including Thai. The Thai-PSQI is equivalent to the original English version when utilized with Thai-speaking patients and has good sensitivity and specificity for identifying good and poor sleepers [30]. All subjects were assessed for sleep quality using the Thai-PSQI [30]. Nineteen items are self-rated, while five additional items may be completed by a roommate or bed partner. The last five questions were collected when a roommate or bed partner was available. These items are intended for clinical information and are not part of the validated PSQI component or global scoring. Because they were not applicable to all participants, they were not included in the primary analysis. The 19 self-rated questions are used to derive seven component scores: sleep duration (DURAT), sleep disturbances (DISTB), sleep latency (LATEN), daytime dysfunction (DAYDYS), habitual sleep efficiency (HSE), sleep quality (SLPQUAL), or sleep medication (MEDS). Each component is scored from 0 to 3, and the seven component scores are summed to produce a global PSQI score ranging from 0 to 21. A global PSQI score ≤ 5 indicates good sleep quality, whereas a score > 5 indicates poor sleep quality. Higher global PSQI scores indicate poorer sleep [3]. The reliability and validity of the Thai-PSQI were evaluated by Sitasuwan et al. (Cronbach’s alpha = 0.837, ICC = 0.89) [30].

### 2.3. Statistical Analysis

All statistical analyses were conducted using IBM SPSS Statistics Version 26 (IBM Corporation, Armonk, NY, USA). The normality of continuous variables was assessed using the Shapiro–Wilk test. PSQI scores were compared among the skeletal groups using the Kruskal–Wallis test, with effect sizes reported as epsilon-squared (ε^2^), calculated as H/(N − 1). Pairwise between-group differences in global PSQI scores were quantified using Hodges–Lehmann location-shift estimates with 95% confidence intervals (CIs). Global PSQI scores were compared between sexes using the Mann–Whitney U test, with effect size reported as *r*, and among BMI categories using the Kruskal–Wallis test, with effect size reported as ε^2^. Associations of age and ANB with global PSQI score were assessed using Spearman’s rank correlation coefficient (ρ). Good and poor sleeper groups were compared using the chi-square test for categorical variables and the Mann–Whitney U test for continuous variables, with Cramer’s V and *r* reported as effect sizes, respectively. Odds ratios (ORs) with 95% CIs for poor sleep were calculated by skeletal pattern using Sk-I as the reference group. Statistical significance was set at α = 0.05.

## 3. Results

### 3.1. Demographic Characteristics

This study included 93 orthodontic patients (32 males and 61 females), with a mean age of 23.68 ± 5.80 years and a mean BMI of 21.09 ± 3.44 kg/m^2^. No statistically significant differences were observed among the three skeletal groups regarding gender, age, BMI, SNA, SN-FH, or FMA. In contrast, SNB, ANB, and Wits appraisal values differed significantly among the groups (Table 1).

### 3.2. The PSQI Scores and Sagittal Skeletal Patterns

The mean global PSQI score for all participants was 6.45 ± 2.85. Overall, 55.9% of participants were classified as poor sleepers based on a global PSQI score > 5. The mean global PSQI scores for the Sk-I, Sk-II, and Sk-III groups were 6.42 ± 2.71, 6.68 ± 3.39, and 6.26 ± 2.46, respectively. No statistically significant difference in global PSQI scores was identified among the three skeletal groups (H = 0.024, *p* = 0.988, ε^2^ < 0.001). The Hodges–Lehmann location-shift estimate was 0 PSQI points for each pairwise comparison: Sk-II vs. Sk-I (95% CI, −1 to 1), Sk-III vs. Sk-I (95% CI, −1 to 1), and Sk-III vs. Sk-II (95% CI, −1 to 1). No statistically significant between-group differences were found for any of the seven PSQI component scores (Table 2).

### 3.3. The PSQI Scores and Demographic Variables

Global PSQI scores did not differ significantly between males and females (U = 843.5, *p* = 0.279, *r* = 0.112). Similarly, no significant difference was found among the BMI categories (<18.5, 18.5–22.9, and 23.0–29.9 kg/m^2^) (H = 0.106, *p* = 0.948, ε^2^ = 0.001). Age was not significantly correlated with global PSQI score (Spearman’s ρ = −0.020, *p* = 0.848) (Table 3).

### 3.4. Characteristics of Good and Poor Sleepers and Associations with Sleep Quality

Among the 93 participants, 41 (44.1%) were classified as good sleepers and 52 (55.9%) as poor sleepers. Poor sleepers accounted for 58.1%, 48.4%, and 61.3% of the Sk-I, Sk-II, and Sk-III groups, respectively. The distribution of good and poor sleepers did not differ significantly among the skeletal groups (χ^2^ = 1.134, *p* = 0.567, Cramer’s V = 0.110). Using Sk-I as the reference group, the odds of poor sleep did not differ significantly for Sk-II (OR = 0.68, 95% CI, 0.25–1.85) or Sk-III (OR = 1.14, 95% CI, 0.41–3.16).

The distribution of gender did not differ significantly between good and poor sleepers (*p* = 0.204, Cramer’s V = 0.132). Similarly, no statistically significant differences were identified between the two sleeper groups for age (*p* = 0.649, *r* = 0.047), BMI (*p* = 0.607, *r* = 0.053), or ANB (*p* = 0.246, *r* = 0.120). In addition, ANB was not significantly correlated with global PSQI score (Spearman’s ρ = −0.032, *p* = 0.763) (Table 4).

## 4. Discussion

The present study compared subjective sleep quality among orthodontic patients with Sk-I, Sk-II, and Sk-III sagittal skeletal patterns. As expected from the classification criteria, ANB angles increased from Sk-III to Sk-I and Sk-II. Although the Sk-II group had the highest mean global PSQI score, followed by the Sk-I and Sk-III groups, the between-group difference was not statistically significant. Overall, 55.9% of participants were classified as poor sleepers based on a global PSQI score > 5; however, the distribution of good and poor sleepers did not differ significantly among the skeletal groups.

These findings are consistent with a previous study [25], which found no significant differences in global PSQI scores among Sk-I, Sk-II, and Sk-III groups in adults seeking orthodontic treatment. However, they reported differences according to vertical skeletal pattern and hyoid-to-SN distance, with lower PSQI scores in the hyperdivergent group than in the normodivergent group and higher scores in participants with a shorter hyoid-to-SN distance. These findings suggest that the sagittal maxillomandibular relationship alone may not capture all craniofacial characteristics relevant to subjective sleep quality. Direct comparison should nevertheless be made cautiously because different ANB thresholds were used, whereas the present study applied Thai-specific reference values.

The potential role of the upper airway remains relevant when interpreting these findings. Previous studies have reported differences in upper airway dimensions according to sagittal skeletal pattern [18,20], with oropharyngeal airway dimensions reported to be largest in Sk-III, intermediate in Sk-I, and smallest in Sk-II [16,18]. The oropharyngeal area has also been reported to correlate negatively with the ANB angle [18,20]. Similarly, one previous cephalometric study reported a higher ANB angle in patients with OSA than in those without OSA [31]. In contrast, other studies found no significant differences in skeletal variables between patients with and without OSA or according to OSA severity [32,33]. When ANB was examined as a continuous measure of sagittal skeletal relationship in the present study, it was not significantly correlated with global PSQI score, and the observed correlation was very weak (Spearman’s ρ = −0.032). Upper airway dimensions were not directly assessed; therefore, the present findings should not be interpreted as evidence that sagittal skeletal morphology is unrelated to airway anatomy. Rather, the correlation between ANB and global PSQI score was very weak in this sample.

Most participants in the present study (88.2%) were younger than 30 years, resulting in a relatively narrow age distribution. Age was not significantly correlated with global PSQI score (Spearman’s ρ = −0.020). Previous studies have reported poor sleep quality and irregular sleep–wake patterns among young adults, with insufficient sleep frequently associated with academic and social demands in undergraduate populations [34,35]. However, these factors were not directly assessed in the present study.

Regarding sex, previous research has reported poorer subjective sleep quality among young adult females than males [36]. In the present study, females had a higher mean global PSQI score than males; however, the difference was not statistically significant, and the observed effect size (r = 0.112) was below the proposed threshold of 0.20 for a small effect [37].

Regarding BMI, although previous studies have reported an association between obesity and poor sleep quality [38,39], no statistically significant difference in global PSQI scores was identified among the BMI categories examined in this study, with a very small effect size (ε^2^ = 0.001). The BMI categories incorporated the WHO underweight threshold of 18.5 kg/m^2^ and the Asian-specific public-health action point of 23.0 kg/m^2^ [40]. Participants with BMI ≥ 30.0 kg/m^2^ and those with physician-diagnosed OSA were excluded according to the predefined eligibility criteria. Although these exclusions were intended to reduce the influence of obesity- and OSA-related factors, they also restricted the range of BMI and sleep-related risk represented in the sample.

No statistically significant differences were identified among the three skeletal groups for any of the seven PSQI component scores, consistent with the findings for global PSQI score. Previous research has reported poorer subjective sleep quality, longer sleep latency, and greater daytime dysfunction among younger adults [41]. Sleep medication use was low across all skeletal groups in the present study, consistent with reports of low sleep-medication use in younger populations [34,35].

Several possible factors may help explain the absence of significant differences in sleep quality among the sagittal skeletal groups. First, sagittal skeletal classification based on ANB reflects only one dimension of craniofacial morphology. Other characteristics, including vertical skeletal pattern, hyoid position, and upper airway morphology, may also be relevant to sleep. Variation in these characteristics within each group may have contributed to the overlap in sleep quality scores and, consequently, to the absence of statistically significant differences among the sagittal skeletal groups. Second, the PSQI measures perceived sleep quality and does not directly assess airway obstruction or sleep-disordered breathing. Therefore, differences in airway anatomy may not always be reflected in global PSQI scores. Finally, sleep quality is multifactorial and may also be influenced by psychological, behavioral, and lifestyle factors that were not assessed in the present study.

Several limitations should be acknowledged. Sleep quality was assessed using the PSQI, a subjective measure, rather than objective sleep measures. Upper-airway dimensions were not directly assessed; therefore, the anatomical pathway proposed to link sagittal skeletal morphology with sleep quality could not be evaluated directly. Although the eligibility criteria were designed to limit the influence of several clinical factors that could affect sleep quality or upper airway characteristics, other potential confounders, including psychological, behavioral, and lifestyle factors, were not assessed, and no multivariable adjustment was performed. Excluding participants with obesity and physician-diagnosed OSA may also have reduced variability in airway-related risk and sleep quality, potentially limiting the applicability of the findings to these populations. Finally, the study was conducted at a single orthodontic center in a predominantly young Thai population, which may limit the generalizability of the findings to other age groups and populations. Future studies should include larger and more diverse samples, direct assessment of upper airway and other craniofacial characteristics, objective sleep measurements such as polysomnography (PSG), and evaluation of relevant behavioral and clinical confounders.

## 5. Conclusions

This study demonstrated no significant differences in subjective sleep quality, as measured by the Thai-PSQI, among patients with different sagittal skeletal patterns in this sample.

## Figures and Tables

**Table 1:** Sample characteristics (n = 93).

Variables	Sk-I (*n* = 31)	Sk-II (*n* = 31)	Sk-III (*n* = 31)	Total (*n* = 93)	*p* Value
Gender					
Male (%):Female (%)	8 (25.8):23 (74.2)	10 (32.3):21 (67.7)	14 (45.2):17 (54.8)	32 (34.4):61 (65.6)	0.263 ^a^
Age (yr)	22.94 ± 4.37	24.55 ± 7.26	23.55 ± 5.49	23.68 ± 5.80	0.913 ^c^
Weight (kg)	54.23 ± 10.44	58.84 ± 11.62	59.26 ± 12.89	57.44 ± 11.79	0.153 ^c^
Height (m)	1.63 ± 0.09	1.65 ± 0.07	1.66 ± 0.09	1.65 ± 0.08	0.352 ^c^
BMI (kg/m ^2^)	20.25 ± 2.63	21.69 ± 3.72	21.34 ± 3.80	21.09 ± 3.44	0.393 ^c^
SNA (°)	84.05 ± 2.67	85.48 ± 3.12	83.61 ± 4.33	84.38 ± 3.50	0.088 ^b^
SNB (°)	80.63 ± 2.68	79.17 ± 3.35	86.18 ± 5.18	81.99 ± 4.89	<0.001 ^c^ *
ANB (°)	3.41 ± 0.95	6.37 ± 0.89	−2.25 ± 2.22	2.51 ± 3.88	<0.001 ^c^ *
SN (mm)					
Total	63.82 ± 2.62	65.01 ± 3.50	66.30 ± 6.11	65.04 ± 4.41	0.152 ^c^
Male	66.01 ± 2.86	68.63 ± 2.69	70.00 ± 6.26	68.58 ± 4.79	0.146 ^c^
Female	63.05 ± 2.10	63.29 ± 2.33	63.25 ± 4.05	63.19 ± 2.80	0.926 ^c^
SN-FH (°)	8.63 ± 1.56	8.33 ± 2.37	8.53 ± 3.08	8.49 ± 2.39	0.835 ^c^
AO-BO (mm)					
Total	0.19 ± 2.55	3.12 ± 2.36	−7.57 ± 4.30	−1.55 ± 5.49	<0.001 ^c^ *
Male	0.9 ± 2.33	4.62 ± 2.94	−9.45 ± 5.33	−2.47 ± 7.54	<0.001 ^c^ *
Female	−0.57 ± 2.56	2.41 ± 1.69	−6.02 ± 2.43	−1.06 ± 4.03	<0.001 ^c^ *
FMA (°)	27.12 ± 7.09	27.71 ± 6.61	24.65 ± 4.98	26.49 ± 6.37	0.134 ^b^

^a^ Chi-square test; data are presented as frequencies (percentages), ^b^ One-way ANOVA and ^c^ A Kruskal—Wallis test were performed for intergroup differences in Sk-I, Sk-II, and Sk-III groups; data are presented as mean ± standard deviation (* *p* ≤ 0.05).

**Table 2:** A comparison of PSQI scores and subcomponent scores.

Variables	Sk-I (*n* = 31)		Sk-II (*n* = 31)		Sk-III (*n* = 31)		Total	H	*p* Value	ε^2^
	Mean ± SD	Median (IQR)	Mean ± SD	Median (IQR)	Mean ± SD	Median (IQR)	Mean ± SD			
1 DURAT	0.77 ± 0.76	1 (1)	0.87 ± 1.06	0 (2)	0.61 ± 0.84	0 (1)	0.75 ± 0.89	1.286	0.526	0.014
2 DISTB	1.16 ± 0.45	1 (0)	1.29 ± 0.46	1 (1)	1.39 ± 0.56	1 (1)	1.28 ± 0.50	3.397	0.183	0.037
3 LATEN	1.26 ± 0.77	1 (1)	1.26 ± 0.82	1 (1)	1.52 ± 0.93	1 (1)	1.34 ± 0.84	1.773	0.412	0.019
4 DAYDYS	1.35 ± 0.84	1 (1)	1.32 ± 0.91	1 (1)	1.10 ± 0.83	1 (2)	1.26 ± 0.86	1.370	0.504	0.015
5 HSE	0.32 ± 0.79	0 (0)	0.52 ± 0.77	0 (1)	0.35 ± 0.71	0 (1)	0.40 ± 0.75	2.648	0.266	0.029
6 SLPQUAL	1.39 ± 0.62	1 (1)	1.29 ± 0.74	1 (1)	1.29 ± 0.64	1 (1)	1.32 ± 0.66	0.908	0.635	0.010
7 MEDS	0.16 ± 0.58	0 (0)	0.13 ± 0.56	0 (0)	0.00 ± 0.00	0 (0)	0.10 ± 0.47	2.912	0.233	0.032
Global PSQI scores	6.42 ± 2.71	6 (3)	6.68 ± 3.39	5 (5)	6.26 ± 2.46	6 (4)	6.45 ± 2.85	0.024	0.988	<0.001

Global PSQI scores and component scores were compared among the Sk-I, Sk-II, and Sk-III groups using the Kruskal—Wallis test. Effect sizes are reported as epsilon-squared (ε^2^), calculated as H/(N − 1).

**Table 3:** Associations between demographic variables and global PSQI scores.

Variables	Group/Category	n (%)	Global PSQI		Test Statistic	*p* Value	Effect Size
			Mean ± SD	Median (IQR)			
Gender	Male	32 (34.4)	6.00 ± 2.27	5.00 (3.00)	U = 843.5	0.279 ^a^	r = 0.112
	Female	61 (65.6)	6.69 ± 3.11	6.00 (4.00)			
Age	Overall	93 (100)	—	—	ρ = −0.020	0.848 ^b^	ρ = −0.020
BMI	<18.5 kg/m^2^	25 (26.9)	6.44 ± 2.97	6.00 (3.00)	H = 0.106	0.948 ^c^	ε^2^ = 0.001
	18.5–22.9 kg/m^2^	43 (46.2)	6.58 ± 3.14	6.00 (4.00)			
	23.0–29.9 kg/m^2^	25 (26.9)	6.24 ± 2.24	6.00 (4.00)			

^a^ Mann–Whitney U test; ^b^ Spearman’s rank correlation; ^c^ Kruskal–Wallis test. Data are presented as n (%), mean ± standard deviation (SD), and median (interquartile range [IQR]), as appropriate. Effect sizes are reported as r for the Mann–Whitney U test, Spearman’s ρ for the correlation analysis, and ε^2^ for the Kruskal–Wallis test. Statistical significance was set at *p* ≤ 0.05.

**Table 4:** Characteristics of good and poor sleepers and associations of skeletal pattern and ANB with sleep quality.

Variables	Good Sleepers PSQI ≤ 5	Poor Sleepers PSQI > 5	*p* Value	Effect Size	OR for Poor Sleep (95% CI)
Gender, n (%)			0.204 ^a^	V = 0.132	—
Male	17 (53.1)	15 (46.9)			
Female	24 (39.3)	37 (60.7)			
Age (years)	23.83 ± 5.74; 22.00 (5.00)	23.56 ± 5.91; 22.00 (5.00)	0.649 ^b^	*r* = 0.047	—
BMI (kg/m^2^)	21.32 ± 3.67; 20.81 (5.13)	20.92 ± 3.28; 20.31 (4.71)	0.607 ^b^	*r* = 0.053	—
ANB (°)	3.05 ± 3.61; 3.70 (6.05)	2.08 ± 4.07; 3.10 (6.50)	0.246 ^b^	*r* = 0.120	—
Skeletal pattern, n (%)			0.567 ^a^	V = 0.110	
Sk-I	13 (41.9)	18 (58.1)			1.00 (reference)
Sk-II	16 (51.6)	15 (48.4)			0.68 (0.25–1.85)
Sk-III	12 (38.7)	19 (61.3)			1.14 (0.41–3.16)
ANB– G lobal PSQI correlation	—	—	0.763 ^c^	ρ = −0.032	—
Total, n (%)	41 (44.1)	52 (55.9)			

^a^ Chi-square test; ^b^ Mann–Whitney U test; ^c^ Spearman’s rank correlation. Data are presented as n (%), mean ± standard deviation (SD), and median (interquartile range [IQR]), as appropriate. Effect sizes are reported as Cramer’s V for chi-square tests, *r* for Mann–Whitney U tests, and Spearman’s ρ for correlation analysis. Odds ratios (ORs) with 95% confidence intervals (CIs) for poor sleep were calculated using Sk-I as the reference group. Statistical significance was set at *p* ≤ 0.05.

## Data Availability

The data supporting the findings of this study are available from the corresponding author upon reasonable request. The data is not publicly available due to privacy and ethical restrictions.
